# “I don’t think I have been out of fight or flight. Ever.” transgender people’s experiences in inpatient psychiatric treatment

**DOI:** 10.1016/j.ijnurstu.2025.105028

**Published:** 2025-02-12

**Authors:** Kristen D. Clark, Jordon D. Bosse, Kasey B. Jackman, David Brown, Jacob Dubay, Jaylyn Jewell, Shea Flanders, Catherine Hardwick, Carol Dawson-Rose

**Affiliations:** a Department of Medical Sciences, Uppsala University, Akademiska sjukhuset, ingang 10, Uppsala, Sweden; b College of Health and Human Services, Department of Nursing, University of New Hampshire, 4 Library Way, Durham, NH, USA; c College of Nursing, University of Rhode Island, 39 Butterfield Road, South Kingstown, RI, USA; d Columbia University, School of Nursing, 560 W 168th St, New York,NY, USA; e New York-Presbyterian Hospital, 560 W 168th St, New York, NY, USA; f Department of Community Health Systems, School of Nursing, University of California San Francisco, 490 Illinois Street, San Francisco, CA, USA

**Keywords:** Hospitals, psychiatric, Transgender persons, Mental health services, Health equity, Mental health, Delivery of health care

## Abstract

**Background:**

Historically, marginalized groups have been deemed unwell and deserving of correction, resulting in disproportionate use of inpatient psychiatric institutionalization. Despite changes over the last hundred years, individuals from marginalized groups continue to experience poor treatment in inpatient psychiatric settings. Transgender people are marginalized in a society where it is assumed that all individuals exist solely as woman or man with predetermined roles influenced by innate biology based on their sex assigned at birth, i.e. gender essentialism. This contributes to mental health disparities (e.g., depression, anxiety, suicidal thoughts, and suicide attempts), which may result in higher acuity symptoms, leading to overrepresentation in inpatient psychiatric settings. Yet, little is known about transgender people’s experiences during inpatient psychiatric treatment.

**Objective:**

To describe the experiences of transgender people in inpatient psychiatric treatment.

**Design:**

A qualitative descriptive study.

**Setting:**

Interviews were held in person or over Zoom.

**Participants:**

Adults who self-identified as transgender and had been admitted to inpatient psychiatric treatment during the last five years were recruited to participate through community organizations, social media, and word of mouth.

**Methods:**

Semi-structured interviews were conducted between March 2019 and June 2022. Data were analyzed using thematic analysis.

**Results:**

Participants (*N* = 15) described experiences within inpatient psychiatric treatment. The first theme, *gender essentialism causes stigmatizing experiences through structural and enacted power*, was characterized by deliberate or accidental misgendering, gender treated as irrelevant to care, pathologized gender diversity, and withholding of gender-affirming needs. The second theme, *psychological and emotional strain as the price paid for enforced gender essentialism*, included examples of drained emotional resources, powerlessness, and worsening of gender dysphoria. Lastly, the theme *actions in disruption of the structural gender essentialist power* illustrated how the gender essentialist systems in place can be interrupted and resisted by transgender patients and healthcare professionals.

**Conclusions:**

Power structures are embedded in psychiatric hospital policies and practices, as well as the physical layout of the hospital, operating under the assumption that all patients are either man or woman based on their sex assigned at birth. Healthcare professionals may unintentionally or deliberately reinforce these structures, further marginalizing transgender patients. Healthcare professionals have the opportunity to disrupt these harmful systems by advocating for and implementing changes that challenge gender essentialism. Creating care environments that incorporate gender diversity allows transgender individuals to focus on their mental health and recovery, rather than expending emotional resources navigating a system that overlooks or invalidates their identities.

**Social media abstract:**

Inpatient psychiatric treatment reinforces gender essentialism, subjecting transgender patients to stigma and mistreatment. Participants described experiences of frequent misgendering, dismissal of gender-affirming needs, and emotional strain from navigating a system designed for non-transgender patients, leading to worse mental health symptoms, including gender dysphoria, and feelings of powerlessness. Healthcare professionals reinforce these harmful systems deliberately or unintentionally. However, instances of disruption by healthcare professionals and transgender participants were observed leading to the creation of affirming experiences despite the prevailing gender essentialism. Future opportunities to disrupt these structures include advocating for systemic change, engaging in patient-centered care, and developing inclusive policies. By creating inpatient psychiatric environments that accommodate gender diversity, healthcare providers could allow transgender patients to focus on their mental health and recovery, rather than combating stigma. Inclusive care can shift the focus from navigating systemic transphobia to healing.

## 1. Background

Inpatient psychiatric treatment has developed substantially from early psychiatric treatment of patients who struggle with their mental health. Institutions, also referred to as sanitariums or asylums, were viewed as a respite for the wealthy and privileged, but were places of unjust confinement, forced labor, and medically sanctioned abuse for others such as those with physical disabilities or who were otherwise deemed socially “undesirable”(Halem et al., 2024; Houston, 2020) as in the case of transgender people (Chicago Medical Society, 1884; Goffman, 1961; Halem et al., 2024; Houston, 2020; Smith, 2021). By the time institutionalization fell out of favor in the late 19th century in the United Kingdom (Houston, 2020) and the mid-20th century in the United States (US; (Davis et al., 2012), the socially vulnerable had been exploited and subjected to innumerable harms such as neglect, physical, emotional, and sexual abuse, and long-term side effects from medical treatments (Dobbing and Tomkins, 2021; Ferleger and Boyd, 1979; Kirkby, 2005; Wipond, 2023).

In the mid-20th century, psychiatry began embracing models of mental illness centered on genetics and neuroscience leading to increasing focus on medication management of psychiatric symptoms (Scull, 2021). Subsequently, new models of care in inpatient psychiatry emphasized the role of the nurse and the therapeutic milieu within inpatient psychiatry (Smith, 2020). These approaches, based on the “Moral Treatment Era” in the United Kingdom (George et al., 2023), combined both the physical and emotional environment with therapies focused on the biological basis of mental illness such as electroconvulsive therapy, neuroleptics, and other medication advances (Hirshbein, 2012; Jones, 1963; Mahoney et al., 2009).

Despite these evolutions in form, the power relations that facilitated the historical abuse of institutionalized patients largely remain in function. The historian and philosopher Foucault describes how society enacts a disciplinary form of power to influence and control human behavior and enforce social norms through carceral means (Foucault, 2020, 2008). Behaviors that are socially deemed undesirable are believed to require suppression and control, which historically included those who were perceived to transgress gender roles, such as one would today describe as transgender people (Chicago Medical Society, 1884; Halem et al., 2024; Manion, 2020). The structural characteristics that enact this institutional form of power remain in place, such as locked units and severely restricted patient autonomy (Jones, 1963; Mahoney et al., 2009). While these measures purportedly ensure safety and create a structured routine as a therapeutic tool, they reinforce the underlying power dynamics of inpatient psychiatric settings, an experience derided widely by patients (Modini et al., 2021; Shields and Davis, 2024; Wood and Alsawy, 2016).

Healthcare professionals, knowingly or unknowingly, participate by enacting institutional power over patients within their care. Inpatient psychiatric hospitals have sought to reduce this perceived power differential through the milieu (Seppänen et al., 2018), such as the use of regular clothing in place of scrubs or white coats for staff, patients leading various day-to-day activities on the unit, and the use of greenspaces. Studies have been conducted to understand how the physical environment impacts patient care, such as its relationship with patient agitation or distress (Jovanovíc et al., 2019; Schaaf et al., 2013; Weber et al., 2022). These perspectives emphasize the milieu as it affects patients’ behavior, placing the responsibility on the patient and omitting the interrelationship with healthcare professionals in contributing to patient agitation, escalation, and how their upholding of structural power is enacted in patient care.

The field of medicine, and healthcare professionals themselves, have historically played a role in socially enforced views of paternalism, eugenics, racial inferiority, white supremacy, heteronormativity, and gender essentialism (Bunton and Petersen, 1997; Drescher, 2010; Horwitz, 2020; Nuriddin et al., 2020). Gender essentialism is the belief, or assumption, that gender is binary, underpinned by biological determinist assumptions that one’s assigned sex at birth determines their gender identity and accompanying social role (LaVoi and Goorevich, 2024). Sexual orientations other than heterosexuality were categorized as a mental illness until as recently as 1973 (Drescher, 2010). “Transsexualism” was added to the DSM in 1980, firmly placing gender diversity in the category of psychiatric pathology and framed through a gender essentialist lens (Crocq, 2021; Drescher, 2010). Efforts to destigmatize gender diversity led to the 1994 revision of the diagnostic term to “Gender Identity Disorder,” which continued to pathologize one’s gender identity. In 2013 the terminology was changed to “Gender Dysphoria,” where the diagnosis criteria emphasized the discomfort or distress experienced due to misalignment of one’s gender and sex assigned at birth instead of one’s gender identity itself (Crocq, 2021). The historical pathologization and social stigmatization of transgender people contribute to explicit and implicit bias within healthcare. Implicit bias toward sexual and gender diversity has been associated with the overdiagnosis/misdiagnosis of transgender people in psychiatry (Bridgwater et al., 2023; Mizock and Lundquist, 2016; Rodriguez-Seijas et al., 2024), as well as the disproportionate prescribing of psychiatric medication when compared to non-transgender people (Carrillo et al., 2023).

Despite efforts to improve inpatient psychiatric environments, evidence of inequalities in treatment and overall care persists, disproportionately so among patients from marginalized groups and children (Hoffmann et al., 2020; Hua et al., 2023; Verbeke et al., 2019). Within psychiatry, the desire for Black individuals to escape enslavement was historically pathologized as a disorder (Hylton, 2024). Implicit bias persists even today. For example, Black adolescents are more likely to be diagnosed with disruptive behavior disorders than attention deficit hyperactivity disorder when compared to white adolescents (Fadus et al., 2020; Stroessner et al., 2023). Recent studies also show that patients from racial and ethnic minority groups are more likely to be assigned to older, lower-quality psychiatric facilities (Michaels et al., 2023), and to be placed in restraints— often for longer duration— than white patients (Singal et al., 2023). Racial and ethnic minority status and lower economic status are also associated with being admitted to inpatient psychiatric facilities with poor safety outcomes (Shields, 2021). Still, little research has explored the experiences of other socially marginalized groups, such as transgender people, who may also face disparities in inpatient psychiatric treatment.

Transgender people’s experiences of poor treatment in a variety of healthcare settings are well documented. However, the majority of research has been dedicated to general healthcare settings, gender-affirming healthcare, or inpatient care related to physical ailments (Ayhan et al., 2019; Cicero et al., 2019; Heng et al., 2018). Thus, less is known about mental healthcare experiences. Studies on transgender people’s experiences in mental health treatment settings describe harassment and reports of inadequate knowledge among healthcare professionals (Clark et al., 2025; White and Fontenot, 2019). However, the majority of these studies focused on the experiences of transgender people in outpatient settings, such as therapy and counseling (White and Fontenot, 2019). There are longstanding disparities in mental health outcomes among transgender people compared to the general population, such as higher rates of suicide attempts and depression (Boyer et al., 2021; Connolly and Gilchrist, 2020; Jackson et al., 2023; Pinna et al., 2022). In the most acute instances, transgender people may receive treatment in inpatient psychiatric hospitals, yet little is known about healthcare experiences in those environments. This limits a broader understanding of the breadth and scope of the experiences of transgender people across the continuum of care, making the exploration of inpatient psychiatric treatment experiences a critical priority. Therefore, the purpose of the present study is to describe transgender people’s experiences in inpatient psychiatric settings. To achieve this, the study was guided by the following research questions: 1) How do transgender people describe their experiences in inpatient psychiatric settings? 2) What shapes these experiences?

## 2. Methods

### 2.1. Study design and setting

A qualitative, descriptive study was conducted to investigate the experiences of transgender people seeking and utilizing inpatient psychiatric treatment. The present study solely examined the data related to transgender people’s experiences during inpatient treatment. This design was chosen for its flexibility, facilitating exploration of the study aim through participants’ lived experiences and elucidating related context (Doyle et al., 2020; Neergaard et al., 2009). Individual, semi-structured interviews were conducted in person in a private meeting space in a library. During the COVID-19 pandemic, recruitment was moved to Zoom to comply with social distancing recommendations. The full details of the methods used in the present study can be found in Boutilier et al. (2024).

### 2.2. Sampling and recruitment

A convenience sample of participants was recruited nationally through community organizations, mental health settings, social network sites, and word-of-mouth. For inclusion, participants had to self-identify as transgender (including nonbinary and other gender expansive identities), be 18+ years old, have been admitted to inpatient psychiatric treatment sometime in the previous five years in the U.S., and be able to understand and communicate in English. Eligible participants were scheduled for a one-on-one, semi-structured interview over Zoom or in person. Twenty individuals were screened and five did not meet inclusion criteria. One excluded participant was under 18 years old and the remainder had last been admitted to inpatient psychiatric treatment more than five years prior. The term “transgender” is used throughout the present study as an umbrella term to encompass all gender identities that are not solely aligned with the societal assumptions placed on a person based on their sex assigned at birth (e.g., transgender woman, nonbinary).

### 2.3. Data collection

Informed consent documentation was provided upon study enrollment and reviewed again before the interview was started. Before beginning the interview, participants were provided the opportunity to verbally consent to participation or decline. A semi-structured interview guide was developed to explore participants’ experiences within inpatient psychiatric treatment using previous literature on the healthcare experiences of transgender people (Cicero et al., 2019; White and Fontenot, 2019) and personal knowledge derived from authors’ clinical practice experience within mental health nursing. The interview guide included broad, open-ended questions designed to elicit rich, detailed descriptions of participants’ experiences while minimizing researcher influence, aligning with the principles of thematic analysis and exploratory qualitative research. The primary question was, “Can you tell me about your time on the inpatient hospital unit?” Probing questions were used to invite elaboration on specific parts of the process, such as the admission, on the unit, and discharge. The full interview guide can be found as a supplement in Boutilier et al. (2024). Interviews lasted approximately 90 min and were conducted from March 2019 to June 2022. Participants were provided a $20 Visa or Mastercard gift card for their time.

### 2.4. Data analysis

Thematic analysis (Braun and Clarke, 2022, 2006) was used to analyze study data in Dedoose software (2021). Guided by a critical realist philosophical approach, line-by-line coding was conducted by two individuals on the research team to capture both participants’ subjective experiences and potential underlying mechanisms or systemic factors shaping these experiences (Braun and Clarke, 2022; Fryer, 2022). Codes were developed inductively, reflecting participants’ descriptions while remaining attentive to how broader institutional, social, or structural contexts might influence these accounts. Disagreements between the study team were resolved by discussion and input by the senior author. Coded data were categorized into themes and representative quotes were selected to exemplify the identified themes or to demonstrate variation within the data.

### 2.5. Positionality and rigor

The authors of the present study like all individuals in society each hold diverse lived experiences that, despite efforts to eliminate bias, may influence the present study. We wish to note that most authors have held positions of power and privilege in the mental health setting through their professional experience providing care for patients, including transgender people, in inpatient and/or outpatient mental health settings. Some authors have personal experiences with mental illness themselves and have received care within inpatient psychiatric settings. Additionally, some authors are transgender people themselves, while some authors are cisgender. Some authors are heterosexual and others are queer. The authors, as a group, hold the belief that transgender individuals have a right to be treated with dignity and respect in all healthcare settings and that transgender people should experience gender-affirming interactions within the healthcare system.

In the present study, we applied several methodological practices that both reduce bias and increase the rigor of qualitative studies. Approaches employed include reflexive and analytic memo-ing, team debriefing, and consultation with outside researchers (Whittemore et al., 2001). Data saturation was assessed through iterative data collection and analysis. By the eleventh interview, no new themes emerged, and consistency in coding was observed across subsequent transcripts (Saunders et al., 2018). Consultations with researchers outside of the study team, including clinicians practicing outside of inpatient psychiatric treatment and individuals who are not transgender, but who have lived experience in inpatient psychiatric settings were conducted as these perspectives are not represented within the study team. The consultation with clinicians practicing outside of inpatient psychiatry helped to ensure our findings could be understood from their role in healthcare. The consultation with individuals who were not transgender but had lived experience of inpatient psychiatric care helped to define the lines where experiences may overlap but underlying processes differed based on social identity. Additionally, member checking (*n* = 3) was conducted to ensure that the analytic results were aligned with participants’ lived experiences and to gain participant insight into the identified themes.

### 2.6. Ethical approval

Ethical approval for the present study was provided by the Institutional Review Board at the University of California San Francisco (IRB 18–25682) and the University of New Hampshire (IRB-FY2021–82).

## 3. Results

### 3.1. Participants

Fifteen transgender adults were interviewed (Table 1), 80 % of whom (*n* = 12) were interviewed over Zoom. The majority of participants were under 30 years of age (*n* = 10) and had at least some higher education (*n* = 12). It was also notable that the majority of participants had experienced more than one psychiatric hospitalization (*n* = 11). Seven participants had been prescribed gender-affirming hormones (estrogen or testosterone) and had been taking them prior to their inpatient psychiatric admission. The majority of these participants (*n* = 5) reported that their prescribed gender-affirming had been withheld at some point during their inpatient hospitalization. For most of them, this lasted the entirety of their admission (*n* = 4).

### 3.2. Thematic overview of qualitative data

**Fig. 1.**
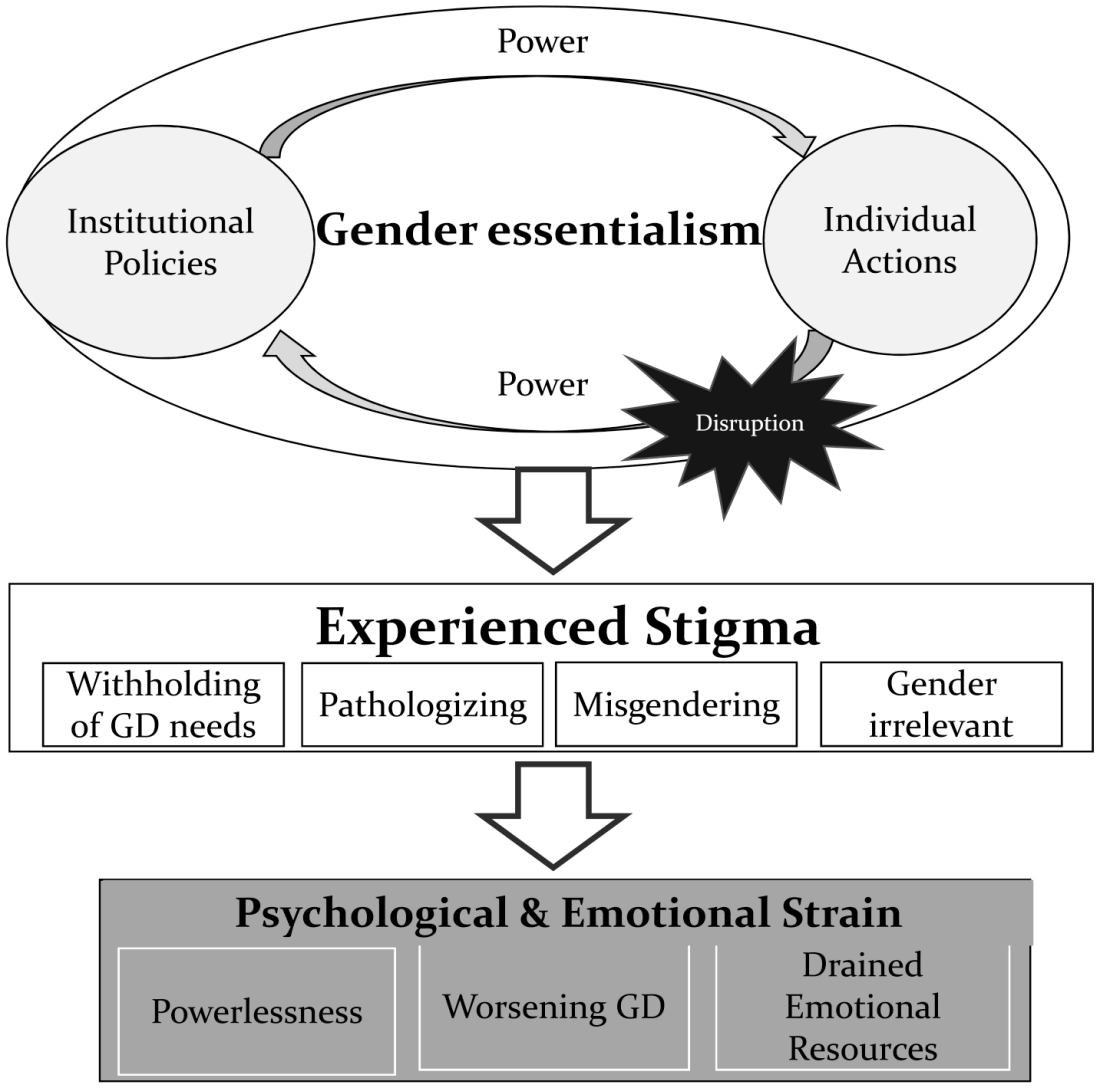
A conceptual model displaying the interrelationships between the main themes and subthemes that describe the experiences of transgender people in inpatient psychiatric treatment settings.

Three overarching themes emerged from the data: 1) *gender essentialism causes stigmatizing experiences through structural and enacted power,* 2) *psychological and emotional strain as the price paid for enforced gender essentialism*, and 3) *actions in disruption of structural gender essentialist power*. Each of the themes, with the main categories of responses, is described below. Representative quotes and where in the treatment cascade the experience occurred (i.e., during admission, on the unit, at discharge) are also described. The interrelationship between these themes is visualized in Fig. 1.

### 3.3. Theme 1: Gender essentialism causes stigmatizing experiences through structural and enacted power

This theme describes how power inherent to institutions and individuals who enact that power, either accidentally or intentionally, is informed by gender essentialism resulting in stigmatizing experiences (Fig. 1). Examples include deliberate or accidental misgendering, pathologized gender diversity, treating gender as irrelevant to care, and withholding gender-affirming needs.

#### 3.3.1. Deliberate and accidental misgendering

Misgendering, or the use of names, pronouns, or other sex/gender-related terms that do not align with a transgender person’s gender and needs, was described as occurring throughout the inpatient psychiatric treatment cascade, starting from the time of admission. Participants described events of misgendering that occurred in ways that were perceived as deliberate or accidental.

Accidental misgendering describes occurrences where the use of incorrect names, pronouns, or other gender-related terms was perceived to be an unintentional mistake, which may or may not have been noticed and corrected by the healthcare professional or peer. These instances of accidental misgendering occurred most frequently as a result of institutional resources or policies, such as healthcare documentation or electronic record keeping that did not communicate the patient’s information in an affirming manner.

“The person at the front desk was nice and they took my insurance and they let me go past. The…ER nurses were really nice. I requested my documents after and I saw they misgendered me everywhere…in the paperwork.”

– Admission, Participant 2, transgender man, Northeastern US.

Deliberate misgendering describes occurrences involving healthcare professionals’ use of the wrong name or pronoun in a manner that communicates disapproval for a transgender person’s gender identity or was perceived as an effort to exert authority in the healthcare space through oppression.

“I straight up told them [healthcare professionals] what my pronouns were and other patients, again, use them [correctly], and I was not the only trans patient …there was a young trans man. He was about 19… and again they were trying to misgender him… it was a very strange experience.”

Admission, Participant 1, nonbinary/transmasculine, Northeastern US.

Participants also described how misgendering occurred on the inpatient units themselves, after admission. However, while on the unit, participants experienced misgendering from other patients in addition to healthcare professionals. When asked about their willingness to share their gender identity and needs related to names and pronouns, one participant stated,

“Oh yeah, if I felt safer. With just the pronouns… and I know it’s hard to control the other patients as well. So … it’s one of those things that I ended up just keeping them [private]. If I was able to be in a position where I was like, ‘oh, I don’t need to keep this to myself… I can be honest with these people.’ I think that would have helped me even more, probably get out even sooner. It was pretty much the same experience each time…, just not feeling comfortable enough, or safe enough, to really be myself”

On the unit, participant 13, nonbinary, Northeastern US.

#### 3.3.2. Gender seen as irrelevant to care

This subtheme describes the experience of participants that their lived experience as a transgender person in society was viewed as irrelevant to their care. This was described most frequently in group therapy settings.

“They [healthcare professional leading group therapy] didn’t use my pronouns, tried to avoid talking gender in group like… Gender issues affected what was going on with me…I really don’t think it was out of malice really. I really think that it was just a lack of understanding on their part.”

On the unit, participant 10, nonbinary, Midwestern US.

Another participant recounted experiencing transphobic behavior by another patient during group therapy. Instead of the healthcare professionals addressing the other patient’s transphobic behavior, the participant was allowed to opt out of therapy sessions, effectively excluding them from participating in their care despite the expectation of attendance placed on other patients.

“It was just another thing I had to deal with. I am generally not a social person. I don’t find it helpful. Even less so when some of the people in said group are transphobic and I’m forced to be around it…, I was allowed to opt out of some therapy groups because of the transphobia….[when probed whether he was asked to not attend group] Yes, because everyone, quote everyone deserves treatment, UN quote.”

On the unit, participant 9, nonbinary/transmasculine, Northeastern US

#### 3.3.3. Pathologizing gender diversity

This subtheme describes experiences where transgender and nonbinary people felt that their gender identity was treated as a problem or symptom of mental illness. Examples of this included the portrayal of gender diversity as imaginary or a distortion of reality.

“There’s a perception that’s coming through …, where the providers are coming from that this [transness or transgender identity] is abnormal, therefore you are abnormal, therefore this is the cause of your mental health issue. There’s a perception that you aren’t actually trans. But they’re… gonna accommodate you in the outside world…, but we’re not gonna accommodate you in mental health [settings].”

On the unit, participant 3, transgender man, Western US.

The pathologization of gender diversity extended to a lack of accessibility to prescribed gender-affirming hormones, such as testosterone and estrogen. Participants described these medications as being withheld during inpatient treatment. They were told that this was either because the medications were assumed to be the cause of the patient’s current mental health crisis or were perceived to potentially worsen it. This is despite all participants who were receiving gender-affirming hormones had done so for a year or longer.

“… in the ER they said that I… would be able to continue testosterone but then the psychiatrist said, ‘We don’t have that here.’ She also said in the paperwork that I saw afterward, she actually unilaterally decided to stop it because she thought it might be worsening the mania… I was just like, I’m already clinging onto my last brain cell trying to please these people and I can’t handle that.”

On the unit, participant 2, transgender man, Northeastern US.

“They [inpatient psychiatrist] wouldn’t give me T [testosterone], cause they decided that… Maybe that’s why I was manic…He’s actually still my psychiatrist. He’s really nice. But he fucked up. He’s apologized but.. it’s 2000 fucking 17… and… they are withholding my hormones… now if all of this research is here, you’re not gonna withhold somebody’s insulin. You’re randomly making this determination because of transphobia.”

On the unit, participant 3, transgender man, Western US.

#### 3.3.4. Withholding of gender-affirming needs

Gender-affirming interventions were described by participants as being withheld during their inpatient psychiatric treatment experiences. Participants described instances where non-medical items, such as chest binders (used by transmasculine people to reduce the prominence of breasts) or razors (used by transfeminine people to remove unwanted hair growth) were denied to participants or restricted as part of the units’ policies and procedures.

“When I kind of accepted my gender dysphoria I tried my best to get rid of…my facial hair by shaving every single day, and trying to shave as close as possible. When I was on the psychiatric ward, I wasn’t allowed to shave… except on specific days, but I missed one of the days, so my facial hair just kept growing, and it made me super uncomfortable. I just, felt like I wanted to rip it all off.”

On the unit, participant 15, transgender woman, Southern US.

Another participant described how their gender identity was ignored when considering room assignments and they were assigned a room based on their sex assigned at birth. They described how this experience contributed to the existing distress surrounding their nonbinary identity in a gender essentialist world that contributed to that specific admission.

“I told them, …that I was nonbinary and that I was struggling with how I could exist in society this way. It’s like they totally disregarded that. I had no one to talk to about it. They put me with a female [roommate]…didn’t ask where I’d be comfortable. That was rough, like because that’s exactly what I was struggling with… can I exist in this world that is so binary-oriented as somebody who doesn’t fit into one of those boxes? …There was nobody that understood what I was going through and that’s why I was in the hospital in the first place.”

On the unit, participant 5, nonbinary/transmasculine, Southern US.

### 3.4. Theme 2: Psychological and emotional strain as the price paid for enforced gender essentialism

This theme describes how, despite patients’ need for emotionally supportive and healing care, gender essentialism enforced through institutional policies and enacted by individual actions resulted in mental harm among transgender participants, such as drained emotional resources, feelings of powerlessness, and worsening of gender dysphoria (Fig. 1).

#### 3.4.1. Drained emotional resources

This subtheme describes the experience of transgender people navigating the need for a high level of care, yet feeling as though they have exhausted their emotional resources. Participants described coping by deciding to “pick their battles” and choose where to engage in correcting mistakes with their name, and pronouns, or speak out against transphobic behaviors.

In regard to misgendering/deadnaming, “*Just because they say like, ‘oh, you know, we’ll make it a point to put it in the notes, but it gets… emotionally exhausting to just keep correcting, and then, that ‘oh I’m sorry’ …. When I’m in there, I’m not really willing to waste more effort correcting someone on my pronouns when I need that energy for other things.”*

On the unit, participant 13, nonbinary, Northeastern US.

Other participants described the emotional exhaustion that arises from anticipating mistakes from healthcare professionals and other patients but still being socially expected to validate the healthcare professional or patient’s discomfort once they realize the mistake.

“This has happened so many times…before I changed my name where the name [chosen name] would be scribbled or something on the form and that’s not the first thing they would be looking at. It’s almost worse than someone just not knowing. [The staff are] coming in and out and you think they know … my name and … my gender. …Then they use my dead name and keep reading and they’re like, ‘oh, oh you prefer to go by [chosen name 1]?’ … It’s very uncomfortable… having to comfort someone who just hurt my feelings.”

- On the unit, participant 11, nonbinary/transmasculine, Midwestern US.

#### 3.4.2. Powerlessness

This subtheme was characterized by experiences when participants felt they could not advocate for themselves or their gender-related needs because of the knowledge that mental healthcare professionals held substantial power over their care and discharge. In some cases, this prevented patients from speaking out against ill-treatment.

“He [psychiatrist] kept asking about my deadname and what I used to be called. I didn’t want to answer. He kept insisting, he said ‘I just want to imagine what you looked like as a girl.’ But what could I say? I felt like, if I didn’t answer him I would be stuck there longer. Like, they would say I was uncooperative and they wouldn’t let me be discharged.”

On the unit, Participant 11, nonbinary/transmasculine, Midwestern US.

“They made me feel like if I didn’t say what they wanted, they would hurt me. So, there was a humiliating interrogation. I had a sibling, who’s nonbinary and they were kind of ignorant about that. This person misgendered me too, this nurse, and I felt really bad because I was like, ‘They’re nonbinary.’ But they kept insisting, like, ‘Are they your sister or your brother?’ I felt like I had to, I said their biological sex and I didn’t want to, but I felt under a lot of duress, like if I don’t [answer], and if they contribute anything I’m saying as a refusal, I’m going to be in trouble.”

Admission, Participant 2, transgender man, Northeastern US.

In other cases, participants perceived their powerlessness when healthcare professionals threatened to withhold gender-affirming treatment to coerce desired compliance.

“…So, somebody said something like, cause I was not wanting them to give me Haldol, and they were like, ‘Well you can’t get your testosterone then.’ …there was some kind of like bargaining thing that I had to do…They were like, ‘If you don’t take this injection then you can’t have the injection you need.’ They asked a question that was [telling me that] I had to say yes to getting [Haldol] injections in order to get my testosterone.”

On the unit, participant 3, transgender man, Western US

#### 3.4.3. Worsening of gender dysphoria

This subtheme reflects participants’ experiences during inpatient psychiatric treatment that heightened feelings of discomfort between their body and gender. Many of these incidents were linked to institutional policies and practices, such as body searches.

“ No one likes being completely naked with nothing on but a paper gown. Least of all somebody who suffers from gender dysphoria. Because then it’s like, there’s nothing separating me from my body. And there’s no way for me to make myself feel more like a woman, and… it’s just degrading.”

Admission, participant 4, transgender woman, Western US.

In other instances, the already triggering requirements were made more so when participants perceived power exerted by healthcare professionals in the form of mocking or demeaning transgender bodies. This is despite the participants “warning” healthcare professionals that their bodies may be different to prevent such occurrences.

“I told the nurse who was doing my admission I was trans so she knew that and then when [performing the body search] she stood way down the hallway and had a security guard standing close to me… a male security guard … usually they do it in a private room like a bathroom or something but, like, she had me leave the door wide open …into a hall where people just enter the unit. I was like completely naked and after I was putting my clothes on she was telling the security guard ‘sorry you had to see that.’ Maybe she wasn’t expecting to see what she saw… I mean I told her I was trans. But she, yeah, she told the security guard, ‘sorry you had to see that’.”

Admission, participant 5, nonbinary/transmasculine, Southern US.

### 3.5. Theme 3: Actions in disruption of structural gender essentialist power

This theme illustrates how the gender essentialist systems and structures in place can be interrupted and resisted by healthcare professionals and transgender patients themselves (Fig. 1).

#### 3.5.1. Disruption by transgender people

Transgender patients described actions they took to disrupt the transphobic behaviors and policies they experienced. Patients described insisting on affirming, appropriate care that aligns with their gender for themselves and other transgender patients on the psychiatric unit despite organization barriers and power dynamics that made such interactions difficult.

“They brought me up to the unit and put me on the women’s side, and I was not OK with that. I was like, ‘What is this? I shouldn’t be on this side.’ They said it was because they didn’t have any have any rooms left on the men’s side, but then I kept telling them like ‘This is not OK. I am not a woman.’ …. They ended up putting me in a room on the men’s side after all, in an empty room by myself…If I didn’t say anything, they probably would have kept me over on the women’s side. ….when I’ve been hospitalized [before], I’ve been so down that I wouldn’t have even said something, but at that time I was able to speak up for myself.”

Admission and On the unit, participant 2, transgender man, Northeastern US.

In other instances, participants described a process of continual adaptation to experiences of misgendering or deadnaming where they would find ways to circumvent structural inadequacies while avoiding direct conflict with healthcare professionals and other patients.

“I asked for name tags. And they’re like ‘well they’re for visitors, but we’ll see what we can find’. And they weren’t real name tags they were just like labels but that’s fine, and so I wrote like my name on it and my pronouns and that helped a lot with people mixing up my name with somebody else. When I was in my room, they would use my dead name. Uhm, because they’d probably just read it on the whiteboard…so then I asked for a sticky note and then I put that under the number for my room like outside the door. I wrote my name and I did some doodling on it to make it fun…”

Participant 12, transgender woman, Northeastern US.

However, this disruption of the gender essentialist systems and structures came with the risk. Participants described fear of being perceived as a “problem” or that their self-advocacy would be perceived as evidence of their psychiatric instability.

“As a patient, knowing that this doctor is treating you poorly… if I call him out on it I will be told that I’m acting inappropriately. That has been true every time… Not only do I have no power but they’re gonna drug me down more and more so I can’t think straight or speak.“

On the unit, participant 3, transgender man, Western US.

#### 3.5.2. Disruption by healthcare professionals

This subtheme describes how healthcare professionals within inpatient psychiatric settings acted to support transgender participants’ needs despite policies or institutional norms. Participants described how some nurses would take on the task of ensuring a patient’s correct name or pronouns were integrated into care by developing workarounds to existing policies and processes or by leading communication with the rest of the healthcare team.

“The nurses were pretty good about it. Like, I told the one nurse my [chosen] name and pronouns. And then she went and told all the other nurses for my room so that when they came in, they weren’t surprised.”

Participant 10, nonbinary, Midwestern US.

However, this disruption by healthcare professionals was less commonly described. It was also limited in its reach and effectiveness by the reliance on specific individuals and the regular changes in staffing each shift. It was also described solely in the context of affirming names and pronouns, contexts that appeared to be of low risk to the healthcare professionals’ status within the hospital organization and with their peers.

## 4. Discussion

This study is among the first to report on the experiences of transgender people in inpatient psychiatric treatment settings. These results convey how transgender individuals’ experiences in inpatient psychiatric settings are shaped by power dynamics rooted in gender essentialism, which led to downstream stigmatizing interpersonal experiences and subsequent psychological and emotional strain. Stigmatizing experiences included *deliberate or accidental misgendering, pathologized gender diversity, treating gender as irrelevant to care*, and *withholding gender-affirming needs*. Even so, participants also described instances where healthcare professionals, or transgender people themselves, acted to interrupt transphobia and advocate for affirming treatment.

Psychiatric systems and structures were historically constructed, as Foucault describes, to enact disciplinary power to control and enforce social norms (Foucault, 2020, 2008). Transgender identities have not only been categorized as undesirable, but ultimately classified as a disorder requiring treatment. Psychiatric systems have been constructed with underlying gender essentialism, resulting in psychiatric systems that are ill-prepared to care for transgender people in an affirming manner. Participants described, for example, experiences of accidental misgendering which appeared to be related to the hospital’s electronic health record systems. Electronic health record systems have been identified as a factor in the stigma experienced by transgender people across healthcare settings as they vary in their ability to record pronouns or changes in name and their usability across healthcare teams to communicate this information (Bosse et al., 2018; Clark et al., 2025). The slow pace of updating systems to accommodate transgender people has been partially attributed to concerns with billing, where health insurance companies have rejected claims because the name, pronouns, or procedure may not match the sex documented in their system (Bakko and Kattari, 2020; James et al., 2016). Similarly, healthcare settings, even as physical structures, have been developed based on gender essentialism. For example, participants described examples of how the psychiatric setting was *withholding of gender-affirming needs*, which resulted in patients being assigned shared rooms based on their sex assigned at birth or a single room on a wing of the hospital based on the same. Previous studies have described how room assignments based on sex assigned at birth may lead to outing (i.e., the disclosure of one’s transgender identity) transgender patients without their consent and making them feel “othered” (Clark et al., 2025). This forms a barrier for transgender people who require acute psychiatric treatment (Boutilier et al., 2024).

In addition to systems and structures, gender essentialism influences care by the very basis of how transgender identities are perceived, predicated by the psychiatric categorization of “gender dysphoria,” a true double-edged sword in this instance. Psychiatric categorization has enabled access to medical interventions for transgender people; it has also influenced the medical field’s perceptions of this population through a gender essentialist, pathologized lens. Transgender people’s gender experience is viewed by the present biomedical paradigm as a “disorder” to be addressed, but at the same time, it remains a “disorder” that healthcare professionals receive limited education on unless they seek specialisation in transgender healthcare (Murray et al., 2025; Sherman et al., 2023; Streed Jr et al., 2024). This positions medical, nursing, and allied health education to treat transgender people as a passing note, leaving personal experience, assumptions, and imaginations to fill in the details (Fisher et al., 2017; Roy and Clark, 2024; Stroumsa et al., 2019). Additionally, the origins of gender dysphoria lie in a binary construction of gender, where someone may describe their gender as woman or man, but this omits the experiences of transgender people where the care needed to affirm their gender may lie elsewhere on the spectrum (e.g., nonbinary or genderfluid identities).

Participants’ descriptions of stigmatizing experiences also exemplified how structural power is enacted and reinforced by healthcare professionals, facilitated by underlying gender essentialism as well. Participants described how *withholding of gender-affirming needs*, such as the use of binders or the inability to shave, was distressing. While this is often a policy enacted to prevent self-harm, gender essentialism can lead healthcare professionals to view these needs as unimportant. Subsequently, potential care adjustments, such as supervised shaving opportunities, are not prioritized or considered unworthy of time and resources. Similarly, hospital policies require that pre-existing medications for patients be reviewed by a psychiatrist or nurse practitioner before they can be continued on the unit. The majority of participants in the present study who were receiving gender-affirming hormones at the time of their inpatient psychiatric hospitalization had them stopped for a prolonged period, or the entirety of their admission. The reason for withholding these medications was often indicative of underlying *pathologization of gender diversity* by healthcare professionals, as gender-affirming hormones were assumed to be the source of their psychiatric symptoms or would make their existing symptoms worse. Yet, this is in direct contradiction to the existing literature demonstrating that gender-affirming hormones are associated with a decrease in psychiatric symptoms, including depression and suicidal ideation (Baker et al., 2021; D’hoore and T’Sjoen, 2022; Shelemy et al., 2024). These examples of structural power enacted by healthcare professionals are evidence of the lack of knowledge held by those working with transgender patients, which in and of itself is a consequence of gender essentialism in medicine, nursing, and other fields of health professional education (Jamal et al., 2024; Kelley, 2024; Nye et al., 2024; Streed Jr et al., 2024; Wagaman et al., 2021).

Some examples of power enacted by healthcare professionals appeared to extend beyond what one could attribute to solely a lack of education. Participants described *deliberate misgendering* and their bodies being treated as a source of shame, leading to a *worsening of gender dysphoria*. One participant described the experience of a body search during admission that was overseen by a security guard rather than unit staff as was the typical practice. The presence of a security guard signals that a transgender person and/or their body is inherently dangerous. Using a security guard for a body search also fails to consider how the presence of someone who looks like a police officer could be activating for a transgender person, given that transgender people are more likely to have negative interactions with the police (Girardi, 2022; Stenersen et al., 2022). Further, keeping the door open to the unit so that others could see into the room does not maintain the transgender person’s dignity or right to privacy. Putting their body on display in this manner exposes the transgender person’s medical history (and body) to others who do not need to know, increasing their vulnerability, and placing them at greater risk of harm from others, including patients (Nordmarken and Reese, 2014; Willging et al., 2019).

Transgender participants described instances where their *gender was treated as irrelevant to care*, where personal comfort and deference were given to a peer who engaged in transphobic behavior during group sessions, thereby using power as healthcare professionals to direct power in a manner that upholds gender essentialism and, subsequently, transphobia (Fig. 1). This is similar to experiences described in other studies, where transgender participants were viewed as attentionseeking or that their experiences in the world related to their gender were irrelevant (Clark et al., 2025). These attitudes have been reflected throughout medicine historically, where the transgression of gender norms was seen as behavior to be corrected through disciplinary power. These dynamics persist as the biological essentialism of gender remains speculative, and the expectations and desires of transgender people regarding their gender expression continue to be questioned within the medical community (Bindman et al., 2022; Halem et al., 2024; Murawsky, 2023).

These stigmatizing experiences, facilitated by structural expressions of power and furthered through enacted power by healthcare professionals, were described to be detrimental to patients’ emotional well-being. Participants described feelings of *worsening of gender dysphoria* and *powerlessness* stemming from their experiences. One participant described how the withholding of their gender-affirming hormones was used as a coercive tool to ensure compliance with another medication. Regardless of the severity of the patient’s psychiatric status in that interaction, one would be hard-pressed to find an example of a healthcare professional withholding blood pressure or thyroid medication in the same manner. The *powerlessness* that transgender people describe feeling in inpatient psychiatric treatment in the face of such experiences is a logical response. Examples such as the participant who was pressured to disclose their deadname by the psychiatrist, an individual with direct control over the discharge plan, underline the power dynamics at play and emphasize to the transgender patient their place within the social hierarchy. The prevailing message from the psychiatric treatment paradigm and those who uphold its authority is clear: the patient is expected to adhere and obey, any behavior otherwise is evidence of insanity and serves as justification for the response from those upholding psychiatric power (Foucault, 2008; Jina-Pettersen, 2022; Johnston and Kilty, 2016).

Underlying gender essentialism and the view that transgender identities are a pathological condition, “not real,” or that transgender people’s gender-affirming needs are not a significant concern summarizes the prevailing attitudes of healthcare professionals as described by participants. Participants described how they subsequently felt *drained of emotional resources*. Instead of being able to focus on healing and recovering from their acute psychiatric symptoms, transgender people expend energy and emotional resources to manage transphobia throughout their hospitalization, risking worsening mental health, continued stigmatizing experiences, and psychological and emotional strain. Transgender participants also highlighted the use of strategies like “picking their battles” to maintain emotional reserves. This is consistent with findings from other studies of healthcare interactions for individuals with marginalized identities (Apodaca et al., 2022). Similarly, participants spoke to the benefits of anticipating being *misgendered* (or otherwise treated poorly), a protective coping strategy that was employed to reduce feelings of being let down when these experiences occurred. While the strategy may be of some benefit to the transgender person when healthcare cannot be avoided (i.e., during crises), expecting rejection is also a salient stressor for transgender people (Rood et al., 2016). Just as experiences of healthcare discrimination can lead to healthcare postponement (Clark et al., 2022), so too, can fears of being treated poorly because of one’s gender identity or expression (Glick et al., 2018b, 2018a). Further, fears of healthcare mistreatment are associated with increases in poor outcomes such as using substances to manage feelings associated with the fear of rejection (Rood et al., 2016), as well as increased odds of reporting depression and past-year suicidal ideation and attempts (Seelman et al., 2017).

Foucault describes how resistance by those who work, or are subjugated, within power structures can act to disrupt them and enact social change (Foucault, 1983). Transgender individuals have long acted in resistance to gender essentialist power structures, particularly in healthcare through community collaboration to establish alternative means of accessing gender-affirming medical treatments and sharing knowledge within the community (Abela et al., 2024). As described in examples of *actions in disruption of structural gender essentialist power*, self-advocacy was necessary to meet their need for gender-affirming environments. They also conveyed the risk these actions posed to their treatment trajectories in inpatient psychiatric treatment, a phenomenon shared in other narratives of healthcare resistance where transgender people were labeled as aggressive, difficult, or otherwise mistreated in response (Abela et al., 2024). Participants also described how the very healthcare professionals who uphold the gender essentialist power structures sometimes played a role to change them in collaboration with transgender people. Collaborations between transgender patients and healthcare professionals hold the potential to impact all levels of care. An evaluation of psychiatric facilities should be conducted to identify where transgender individuals could face obstacles in a therapeutic inpatient environment, such as room assignments and bathroom organization (Britt-Thomas et al., 2023). There has recently been a call to focus efforts on reimagining systems of care delivery based on transgender peoples’ lived experiences, which enhances both hospital engagement and community connection by including patients as decision-makers (Kia et al., 2023). This can be implemented broadly as new healthcare environments are planned and remodeled. It can also be implemented in existing psychiatric hospitals. For example, transgender people being admitted should be involved in decision-making whenever possible regarding room assignments and bathroom access to communicate what feels safest and most comfortable for their specific circumstances.

Electronic health record systems should be evaluated for their existing capacity to include chosen names and pronouns (Grutman, 2023), and if this is not possible, a unit protocol can be developed to ensure continuity of affirming care (Cicero et al., 2023). Documentation should be consistent with the patient’s gender identity. Misgendering in healthcare records, when discovered after the fact, can perpetuate mistrust of not only providers but the healthcare system as a whole (Alpert et al., 2023). Safety protocols, such as restrictions on clothing and access to shaving are sometimes necessary but should be evaluated on a case-by-case basis with the understanding that allowing transgender people to express their gender in an affirming manner is a mental health intervention in and of itself (Cicero et al., 2023). Similarly, gender-affirming hormones are a medically necessary medical treatment and should be viewed as such (Coleman et al., 2022; Endocrine Society, 2020; World Medical Association, 2024). Depriving transgender people of medically necessary medication is directly harmful to mental health outcomes and should be only done when absolutely necessary and rooted in scientific evidence. When the psychiatric facility does not have immediate access to gender-affirming hormones, patients should be allowed to use their personal supply, an alternative that can be easily accommodated as it is already part of many institutional policies for other less frequently carried medications. Additionally, ensuring that there are clear policies in place to support the inclusive care of transgender people and that healthcare professionals are educated on their existence, purpose, and implementation is necessary. Inpatient psychiatric hospitals often do not have policies related to caring for transgender people in place, and among those that do, their implementation is unclear or inconsistent (Britt-Thomas et al., 2023). However, without the participation of transgender people in these processes, healthcare institutions will continue to reinforce cisnormative power structures as they cannot be dismantled without the participation of those who they oppress.

### 4.1. Limitations and strengths

These findings underscore the ways in which seeking mental health care has the potential to cause harm to transgender patients through systemic transphobia and enforcement of gender essentialism in the ways power is exerted over patients through institutional policies and individual behaviors. However, there remain notable limitations. This study is cross-sectional and, therefore, causal relationships cannot be inferred. The sample is small but given the richness and detail in the data collected and the saturation of themes, the sample size was sufficient to provide meaningful insights. It is however homogenous in that the sample is entirely from the U.S. and predominantly white. The shift from in-person interviews to solely Zoom interviews as a result of the COVID-19 pandemic may have posed a barrier for the participation of individuals who lacked access to the necessary devices, internet service, or private space.

Outside perspectives from external sources including researchers outside of inpatient psychiatry and individuals with lived experience in inpatient psychiatric settings but who were not transgender were included. However, while several members of the team are transgender, external perspectives from individuals in the transgender community were not sought. This could have impeded the development and description of the results. Individuals with multiple marginalized groups, such as racial and ethnic minorities and sexual minorities (e.g., lesbian, gay, bisexual), may have experiences in inpatient psychiatric settings that vary from the findings described in the present study. Future research should examine these experiences at the intersections of multiple marginalized identities. Despite these limitations, our findings underscore the ways transgender individuals may face harm during inpatient psychiatric treatment through systemic transphobia, with significant power exerted over them through the enforcement of gender essentialism in both institutional policies and individual behaviors—such as those from staff and other patients.

## 5. Conclusions

The findings of the present study describe the experiences of transgender people in inpatient psychiatric treatment as stemming from *gender essentialist power structures that lead to stigmatizing experiences* and *psychological and emotional strain as the price paid* during inpatient psychiatric treatment. These power structures are enacted by healthcare professionals through institutional policies and the hospital itself, as it is built under gender essentialism and, thus, struggle to accommodate gender diversity. Consequently, policies and structures are then accidentally, or intentionally, enacted by healthcare professionals and become tools where personal biases and transphobia are used against transgender patients. Ultimately, healthcare professionals can seek to disrupt these power structures by advocating for and establishing systems of change to prevent gender essentialism from enacting further harm to transgender patients. This was described in the present study as the theme, *actions in disruption of structural gender essentialist power.* Disruption can be accomplished through changes in healthcare professionals’ practice, seeking patient-centered interactions with transgender people, and engaging in the development of inclusive policies within their psychiatric inpatient setting. Creating environments where gender diversity is incorporated into systems and the provision of care can allow transgender people to invest emotional resources in healing and recovery from their acute psychiatric condition.

## Figures and Tables

**Table 1:** Characteristics of transgender and nonbinary participants who have been admitted to inpatient psychiatric care within the past 5 years.

ID	Gender Identity	Sexual Orientation	Race/ Ethnicity	US Region	HRT Withheld
1	Nonbinary/Transmasculine	Pansexual/Queer	Biracial/Hispanic	Northeast	No
2	Transgender Man	Queer	Multiracial	Northeast	Yes
3	Transgender Man	Straight/Heterosexual	White	West	Yes
4	Transgender Woman	Queer	White	West	Yes
5	Nonbinary/Transmasculine	Gay	White	South	Yes
6	Transgender Woman	Bisexual	White	Northeast	No
7	Nonbinary	Bisexual	Asian	Midwest	n/a^a^
8	Transgender Woman	Asexual	White	Midwest	n/a^a^
9	Nonbinary/Transmasculine	Homoromantic	White	Northeast	n/a^a^
10	Nonbinary	Asexual	White	Midwest	Yes
11	Nonbinary/Transmasculine	Bisexual	White	Midwest	n/a^a^
12	Transgender Woman	Queer	White	Northeast	n/a^a^
13	Nonbinary	Pansexual	Latine/x	Northeast	n/a^a^
14	Nonbinary	Homoflexible	White	Midwest	n/a^a^
15	Transgender Woman	Bisexual	White	South	n/a^a^

a n/a indicates that the participant was not prescribed gender-affirming hormone therapy at the time of inpatient treatment

## Data Availability

The data for this study are not publicly available due to privacy concerns. However, the data are available from the corresponding author upon reasonable request. Requests for data will be considered on an individual basis, ensuring that any sharing complies with the ethical guidelines and privacy protections agreed upon by the participants.

## What is already known

- Transgender and nonbinary people have high rates of poor mental health.
- Many transgender and nonbinary people report mistreatment and rejection in healthcare settings.

## What this paper adds

- This study describes transgender and nonbinary people’s experiences during inpatient psychiatric treatment, illuminating how a treatment process that is intended to provide respite and healing instead enacts harm.
- Gender essentialism informs psychiatric systems and can lead to stigmatizing experiences for transgender and nonbinary patients that result in emotional and psychological harm.
- Healthcare professionals working in gender essentialist systems have opportunities to interrupt and enact meaningful change, particularly when in partnership with transgender and nonbinary people.
